# Advanced Molecular Characterization Using Digital Spatial Profiling Technology on Immunooncology Targets in Methylated Compared with Unmethylated IDH-Wildtype Glioblastoma

**DOI:** 10.1155/2021/8819702

**Published:** 2021-02-24

**Authors:** H. Barber, A. Tofias, B. Lander, A. Daniels, J. Gong, Y. Ren, X. Ren, Y. Liang, P. White, K. M. Kurian

**Affiliations:** ^1^Brain Tumour Research Centre, Bristol Medical School, University of Bristol, Bristol, UK; ^2^NanoString Technologies, Inc., Seattle, WA, UK; ^3^University of West of England, Bristol, UK

## Abstract

**Introduction:**

Glioblastoma (GBM) is the most common primary adult brain tumour with a median overall survival (OS) of 12–15 months. Molecular characterization of multiple immunooncology targets in GBM may help target novel immunotherapeutic strategies. We used NanoString GeoMx® Digital Spatial Profiling (DSP) to assess multiple immunooncology protein targets in methylated versus unmethylated IDH-wild-type glioblastoma.

**Methods:**

NanoString GeoMx® DSP technology uses multiple primary antibodies conjugated to indexing DNA oligos with a UV photocleavable linker. Tissue regions of interest (ROIs) are selected with bound fluorescent antibodies; oligos are released via a UV-mediated linker and quantitated. We used DSP multiplex analysis of 31 immunooncology proteins and controls (CD4, CD14, CD68, CD8A, B7-H3, PD-L1, CD19, FOXP3, CD44, STAT3 (phospho Y705), CD45, Pan Cytokeratin, MS4A1/CD20, CD45RO, PD1, CD3, beta-2 microglobulin, VISTA, Bcl2, GZMB, PTEN, beta-catenin, CD56, Ki-67, STAT3, AKT, p-Akt, S6, Histone H3, IgG Rabbit control, and Mouse IgG control) from ROIs in a cohort of 10 IDH-wild-type glioblastomas (5 methylated and 5 unmethylated). An nCounter platform allowed quantitative comparisons of antibodies between ROIs in MGMT methylated and unmethylated tumours. Mean protein expression counts between methylated and unmethylated GBM were compared using technical and biological replicates.

**Results:**

The analysis showed 10/27 immunooncology target proteins were significantly increased in methylated versus unmethylated IDH-wild-type glioblastoma tumour core (false discovery rate (FDR) <0.1 by Benjamini–Hochberg procedure).

**Conclusions:**

NanoString GeoMx® DSP was used to analyse multiple immunooncology protein target expression in methylated versus unmethylated IDH-wild-type glioblastoma. In this small study, there was a statistical increase in CD4, CD14, CD68, CD8A, B7-H3, PDL-1, CD19, FOXP3, CD44, and STAT3 protein expression in methylated versus unmethylated GBM tumour core; however, this requires larger cohort validation. Advanced multiplex immunooncological biomarker analysis may be useful in identifying biomarkers for novel immunotherapeutic agents in GBMs.

## 1. Introduction

Glioblastoma (GBM), the most common primary malignancy in adults, has an annual incidence of 3.2 per 100,000 [1]. Despite advances in surgical resection, chemotherapy, and radiotherapy, GBM has a very poor prognosis [2]. Immune checkpoint inhibitors (ICIs) have shown efficacy in several cancers, including, and in particular, melanoma, head and neck cancer, renal cell carcinoma, non-small-cell lung cancer, and colorectal cancer [3–7]. ICIs, such as nivolumab and pembrolizumab, act to increase the immune response to an individual tumour by blocking the PD-1/PD-L1 interaction between host and tumour which may in part inhibit a T-cell-mediated immune reaction and allow the tumour to evade the normal protective immune response [8]. Several clinical trials are underway to investigate the use of ICIs to enhance the immune response in GBM [9]. In particular, CheckMate 143, a randomized phase 3 clinical trial evaluating the efficacy and safety of PD-1 inhibitor nivolumab *Opdivo* in patients with first recurrence of glioblastoma multiforme (GBM), did not meet its primary endpoint of improved overall survival over bevacizumab monotherapy highlighting the need for more detailed analysis of immunooncology biomarkers within GBM in order to better target novel immune therapeutics [10, 11]. Previous work in a cohort of 135 GBMs detected PD-L1 gene expression and Tumour Infiltrating Lymphocytes (TILs) in the majority of GBM cases suggesting potential utility of ICIs, although PD-L1 expression and TILs density were shown to be unrelated to GBM outcome and methylation status was not well defined [12].

Protein expression in formalin-fixed, paraffin-embedded (FFPE) tissue is conventionally measured by immunohistochemistry or immunofluorescence on a small number of proteins. By contrast, novel NanoString Digital Spatial Profiling (DSP) technology allows highly multiplexed and spatially resolved analysis of protein or RNA targets in multiple of Regions of Interest (ROIs) in FFPE tissue. With the theoretical ability to determine up to 800 protein targets on one FFPE section down to single-cell resolution, multiple analytes, including immune targets, can be measured simultaneously using an optical-barcode-based platform [13]. Furthermore, this technology enables multiple ROIs to be analysed, increasing the likelihood of identifying and locating variations in targets across the tissue due to intratumour heterogeneity [14].

We used NanoString GeoMx® Digital Spatial Profiling (DSP) technology in 10 cases of IDH-wild-type glioblastoma [15] to quantify 27 immunooncology protein targets between ROIs in MGMT methylated and unmethylated tumours, additionally comparing tumour core and invasive margin regions.

## 2. Materials and Methods

**Figure 1:**
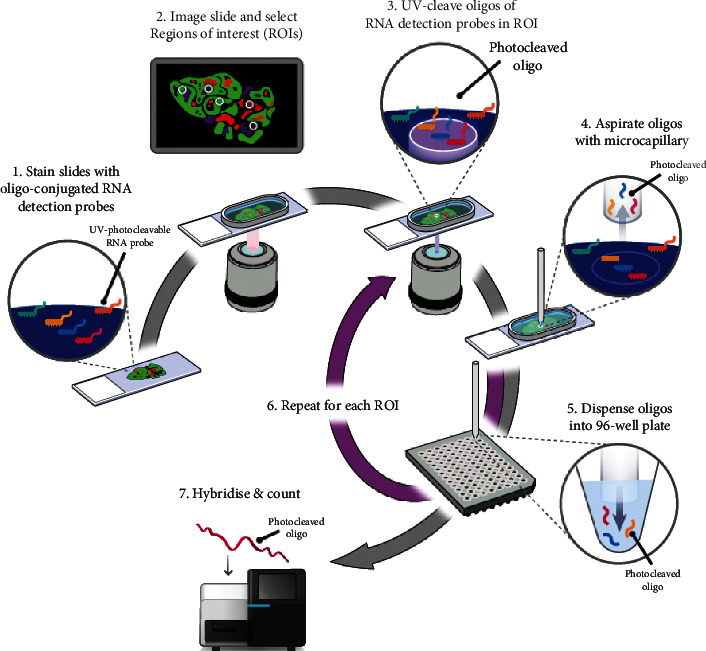
Digital Spatial Profiling workflow. Morphology markers (GFAP, DAPI, and CD3) plus a high-plex oligo-labelled antibody cocktail were first applied to the section. Regions of Interest (ROIs) were then selected for high-plex profiling using visible wavelength low-plex imaging to establish the tumour “geography.” Ultraviolet was used to release the oligo tags at the selected ROIs. The released tags were stored in a microtiter plate, which was then indexed and hybridized to barcodes. Up to 1 million data points per ROI were digitally counted and this data was analysed with nSolver™ Advanced Analysis Software (NanoString, 2018).

DSP (see Figure 1) was undertaken using a cocktail of primary antibodies (NanoString GeoMx® Digital Spatial Profiling [DSP] technology immune panel Table 1) each with a unique, UV photocleavable indexing oligo. The tissue slide was placed on the stage of an inverted microscope. A custom gasket was then clamped onto the slide, allowing the tissue to be submerged in 1.5 mL of buffer solution. The microcapillary tip was connected to a syringe pump primed with buffer solution, allowing for accurate aspiration of small volumes (<10 *µ*L). Under the microscope, wide-field fluorescence imaging was performed with epi-illumination from a visible LED light engine. The ROI was then located using fluorescence imaging. A 20x image corresponded to 650 *µ*m × 650 *µ*m of tissue area using a Complementary Metal Oxide Semiconductor (CMOS) camera. The composite 20x images provided a high-resolution image of the ROI. The ROIs were then selected based on the fluorescence information and sequentially processed by the microscope automation.

The steps performed for each ROI by the microscope automation were as follows. First, the microcapillary tip was washed by dispensing clean buffer out of the capillary and into a wash station. Next, the tissue slide was bulk washed by exchanging the buffer solution on the slide via the inlet and outlet wash ports on the gasket clamp. The microcapillary tip was then moved into position directly over the ROI with 50 *µ*m from the tissue. The local region of tissue around the ROI was washed by dispensing 100 *µ*L of buffer solution from the microcapillary. Then, the area of tissue within the ROI was selectively illuminated with UV light to release the indexing oligos. UV LED light was collimated to be reflected from the Digital Mirror Device (DMD) surface into the microscope objective and focused at the sample tissue. Each micro mirror unit in the DMD corresponded to ∼1 *µ*m^2^ area of sample and reflects the UV light in a controlled pattern based on the ROI selection in the image. Following each UV illumination cycle, the eluent was collected from the local region via microcapillary aspiration and transferred to an individual well of a microtiter plate/strip tube. Once all ROIs were processed, indexing oligos were hybridised to NanoString GeoMx® Digital Spatial Profiling (DSP) technology's optical barcodes for ex situ digital counting and subsequently analysed with an nCounter® Analysis System.

### 2.1. Case Selection

Full ethical approval from Brain Tumour Bank South West (REC: 11/WA/0016) was obtained for this study. In total, 10 IDH-wild-type glioblastoma cases (5 MGMT methylated and 5 MGMT unmethylated), were selected from patients diagnosed between 2012 and 2013. MGMT methylation status had been previously determined by the Bristol Genetics Laboratory by the use of the pyrosequencing assay described by Dunn et al. with optimised cut-offs: ≥12% methylated and ≤11% unmethylated (CpG sites 72–83) [16]. All slides were reviewed by a certified neuropathologist (KMK) to confirm the diagnosis. For each specimen, one 4‐*μ*m‐thick FFPE section was cut from a single representative block and collected on adhesive glass slides. Each of the 5 slides contained an adjacent MGMT methylated and unmethylated GBM section. Samples were classified as margin (≤30% in cellularity of tumour cells compared to background reactive neuroglial tissue) and solid tumour core (≥80% in cellularity of tumour cells compared to background reactive neuroglial tissue), in order to make clear distinction between the infiltrating edge and solid centre of the tumour.

### 2.2. ROI Selection

**Figure 2:**
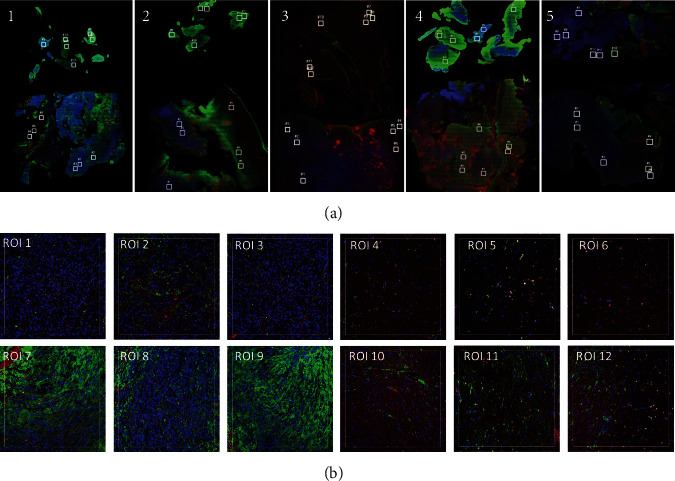
(a) Photomicrographs of selected cases of glioblastoma (1–5). Each slide had a total of 12 regions of interest (ROIs) selected, denoted by white square: ROIs 1–3 (tumour-MGMT unmethylated), ROIs 4–6 (margin-MGMT unmethylated), ROIs 7–9 (tumour-MGMT methylated), and ROIs 10–12 (margin-MGMT methylated). (b) Close-up of regions of interest 1–12 selected on slide 1. The sections were stained with the visualization markers CD3 (red), GFAP (green), and DNA (blue). ERCC normalised data is shown below.

The sections (see Figure 2) were stained with the visualization markers CD3 (red), GFAP (green), and DNA (blue) to assist with the selection of ROIs. Six ROIs per section were selected by aligning fluorescent images with H&E images with ROIs predetermined by a certified neuropathologist (KMK). Blood vessel and necrosis were kept to a minimum in the selected ROI.

Each slide contained the following ROIs; denoted by white square (Figure 2): ROIs 1–3 (tumour core-MGMT unmethylated), ROIs 4–6 (tumour margin-MGMT unmethylated), ROIs 7–9 (tumour core-MGMT methylated), and ROIs 10–12 (tumour margin-MGMT methylated).

### 2.3. Processing

DSP enables detection of multiplexed proteins from the surface of FFPE tissue and protein quantitation from a defined ROI. The cocktail of 31 antibodies (including controls) was applied to each FFPE unstained section. Each antibody was conjugated to an indexing oligo by a UV-cleavable linker. Upon highly controlled UV exposure of individual ROIs by a programmable micromirror device, the oligos were released by photocleavage and could be removed using a microcapillary tip into a microtiter plate [13]. Following each UV cycle, the oligos were subsequently hybridised to NanoString GeoMx® Digital Spatial Profiling (DSP) technology's “barcodes” and can be processed by quantitative detection using the nCounter analysis system providing digital counts [17] (Figure 1). The digital counts produced corresponded to the abundance of each targeted protein in the ROI.

## 3. Data Quality Control

nCounter assays included a set of 6 positive and 6 negative hybridization control probes, positive (POS) and negative (NEG) A–F, respectively, to monitor hybridization efficiency, prep-station purification, and imaging. POS control targets were built into the Code Set reagent and thus reflected systematic variability between the assays.

POS control performance was determined by the linearity of counts versus protein tag set concentration. Protein tag concentrations in the hybridization for protein tag positive control targets ranged from 128 fM (POS_A) to 0.125 fM (POS_F) in a 4-fold titration. The expected correlation of POS control counts to protein tag target concentration was *R*2 > 0.95.

Three data normalization systems were determined.

### 3.1. Positive Protein Tag Normalization (ERCC Normalization)

Data was normalised using the geometric mean of the protein tag positive control in each sample. For optimal results, normalization factors ranged between 0.7 and 1.7 for all assays (approximately 1.0 is optimal normalization factor).

### 3.2. Housekeeper Normalization

S6 ribosomal protein and histone 3 were included in the antibody panel as housekeeper proteins.

### 3.3. Signal:Noise Normalization

Mouse IgG1 and Rabbit IgG isotype controls were included in the antibody panel to assess the signal-to-noise ratio.

Area normalization was not applicable for this study; all ROIs were selected with the same area.

## 4. Statistical Analyses

### 4.1. Preprocessing

Four ROIs (three in one sample and one in another sample) were excluded due to low housekeeping gene count (histone H3 <1000). Housekeeping normalization factors were calculated by dividing the geometric mean of histone H3 and ribosomal S6 of individual ROI by the mean of all ROIs. The raw count was divided by the normalization factor. The normalised data was log2 transformed for variance stabilization.

### 4.2. Comparing Methylation Status

To compare the expression of genes between methylation status, the log2 normalised count was fit by the following linear mixed effect model: Log2 normalised count ∼ methylation + (patient: methylation).

Methylation is the fixed main effect that assesses the overall difference. The random interaction effect between patient and methylation in the parentheses allows the effect of methylation to vary among patients. The model is fit with the R package lmerTest. Log2 fold change was estimated and the *p* value was inferred using Satterthwaite's degrees of freedom method provided in lmerTest. The analysis was done for tumour ROI and margin ROI separately.

## 5. Results

The mean normalised count values for all 27 immune protein targets ranged from 6.47 to 19.26 (see Table 1: mean (SD) log 2 housekeeping normalised values by location (core versus margin) and MGMT status). Overall, there was no statistical difference in counts between tumour core and tumour margin in either MGMT methylated or MGMT unmethylated IDH-wild-type-GBM cases (Table 1). However, there was a statistically significant increase in CD4, CD14, CD68, CD8A, B7-H3, PDL-1, CD19, FOCP3, CD44, and STAT3 protein expression in methylated versus unmethylated GBM tumour core (see Table 2). In the following, mean [SD] are reported.

### 5.1. Immune Cell Biomarker Counts (Table 1)

We observed comparatively low counts of CD45 pan-leukocyte marker (MGMT unmethylated tumour core mean normalised value (MGMTuc) = 7.93 [0.83], MGMT methylated tumour core mean normalised value (MGMTmc) = 9.19 [1.00]), and CD45RO isoform (MGMTuc = 7.48 [0.50] and MGMTmc = 8.29 [0.81]) which is in keeping with low immune infiltration irrespective of MGMT methylation status within IDH-wild-type GBM. The mean normalised count values of T lymphocyte marker CD3 are low (MGMTuc = 8.04 [1.02] and MGMTmc 8.90 [0.61]) and T-helper cell marker CD4 (MGMTuc = 9.58 [1.01] and MGMTmc = 11.22 [0.65]), which may indicate very low levels of T-cells and T helper cells, respectively. Both B-cell markers CD20 (MGMTuc = 6.47 [0.53] and MGMTmc = 7.26 [0.63]) and CD19 (MGMTuc = 6.66 [0.63] and MGMTmc = 7.77 [0.74]) are reduced which also may indicate a very low number of B-lymphocytes within cases. Mean normalised values for cytotoxic T-cell marker CD8A (MGMTuc = 6.90 [0.66] and MGMTmc = 8.30 [0.78]) may indicate low levels of cytotoxic T cells. By comparison, GZMB (the protease granzyme B values = MGMTuc = 10.27 [0.60] and MGMTmc = 10.65 [0.47]) shows elevated values. GZMB is a serine protease commonly found in the granules of cytotoxic T-cells and NK cells, and although difficult to be certain, the mild increased values may represent a difference in NK cells. We observed comparatively low normalised mean values of PD-1 (MGMTuc = 7.43 [0.44] and MGMTmc = 8.08 [0.73]) in keeping with overall comparatively low T-cell-associated proteins in our cohort.

### 5.2. Immune Checkpoint and Cancer Biomarker Counts (Table 2)

Table 2 shows *p*-values for MGMT comparison in tumour core (column 2) and margin (column 3) and for comparing core against margin in methylated (column 4) and in unmethylated (column 5). Overall there was a statistically significant difference between methylated and unmethylated tumour cores, but not tumour margins. GeoMx DSP shows 10/27 immunooncology target proteins were significantly increased in methylated versus unmethylated IDH-wild-type glioblastoma tumour core but not margin (false discovery rate (FDR) <0.1, by Benjamini–Hochberg procedure). In particular, there was a statistically significant increase in CD4, CD14, CD68, CD8A, B7-H3, PDL-1, CD19, FOXP3, CD44, and STAT3 protein expression in methylated versus unmethylated GBM tumour core (see Table 2). There was no statistically significant difference in PD-1, VISTA, CD45, CD45RO, CD3, CD20, GZMB, STAT3 (phospho Y705), beta-2 microglobulin, CD56, beta-catenin, AKT, P-AKT, PTEN, Bcl2, Pan Cytokeratin, and Ki-67 protein expression.

In particular, in our cohort, checkpoint biomarker PD-L1 is statistically increased in MGMT methylated tumour cores compared with unmethylated tumour cores of GBMs (*p* = 0.025) (see Table 2). The checkpoint molecule B7-H3 (CD276) is statistically increased in MGMT methylated tumour cores compared with unmethylated tumour cores of GBMs (*p* = 0.023); MGMT methylated tumour core counts: 14.07 [1.02] compared to MGMT unmethylated GBM (tumour core: 12.35 [0.87]). V-domain immunoglobulin suppressor of T-cell activation VISTA is homologous to PD-L1 and a suppressor of T-cell activation, synergising with PD-1. By contrast, VISTA is not statistically increased in MGMT methylated tumour cores compared with unmethylated tumour cores of GBMs (*p* = 0.153); MGMT methylated tumour core (11.94 [0.70]) and MGMT unmethylated tumour core (11.42 [0.77]) although mean normalised counts are increased.

## 6. Discussion

In this paper, we used NanoString GeoMx® Digital Spatial Profiling (DSP) technology to analyse multiple immunooncology targets in methylated against unmethylated IDH-wild-type GBM tumours. NanoString GeoMx® Digital Spatial Profiling (DSP) technology has the theoretical ability to analyse a potential of 800 protein targets, therefore allowing in-depth analysis of panels of tissue-based prognostic and predictive biomarkers, in-depth monitoring of response to therapies, and more rapid identification of targets of disease. The small sample of this technology feasibility study requires further validation of results in larger cohorts.

### 6.1. Immune Infiltration

The mean log 2 housekeeping normalised values by location and MGMT status for all 27 immune protein targets ranged from 6.47 to 19.26 in our study (Table 1). NanoString GeoMx® Digital Spatial Profiling (DSP) technology has been tested in several studies reporting its robustness, sensitivity, and reproducibility [18–20]. However, one potential disadvantage we identified using the technique was the lack of ability to draw direct comparisons between count values and immune cell numbers within samples because it is not possible to visualise cells and also there was a complex normalization process and no standardised normalised tables were available for comparison. For example, the mean normalised values of CD3 (MGMTuc = 8.04 [1.02] and MGMTmc = 8.90 [0.61]) and CD4 (MGMTuc = 9.58 [1.01] and MGMTmc = 11.22 [0.65]) may indicate very low levels of T-cells and T-helper cells, respectively, within the tumours (see Table 2). In turn, both CD20 (MGMTuc = 6.47 [0.53] and MGMTmc = 7.26 [0.63]) and CD19 (MGMTuc = 6.66 [0.63] and MGMTmc = 7.77 [0.74]) may indicate a low number of B lymphocytes. Mean normalised values for CD8A (MGMTuc = 6.90 [0.66] and MGMTmc = 8.30 [0.78]) may indicate low levels of cytotoxic T cells in the specimens studied; however, by comparison, GZMB (the protease granzyme B values = MGMTuc 10.27 [0.60], MGMTmc 10.65 [0.47]) showed comparatively elevated values. GZMB is a serine protease commonly found in the granules of cytotoxic T-cells and NK cells, and although difficult to be certain, it raises the possibility of increased NK cells within these specimens. These findings however are in keeping with previous work in malignant gliomas suggesting that B-cells and the subtypes of T-cells are found in these tumours to varying degrees, with particularly low numbers of B-cells and CD4 helper T-cells [21]. Here, we observed comparatively low values for CD45RO (MGMTuc = 7.48 [0.50] and MGMTmc = 8.29 [0.81]) and CD45 (MGMTuc = 7.93 [0.83] and MGMTmc = 9.19 [1.00]), which is in keeping with low immune infiltration irrespective of MGMT methylation status. Previous work has also suggested sparse-to-moderate density of TILs in 85 of 117 (72.6%) IDH-wild-type glioblastoma specimens (CD3+ 78/117, 66.7%; CD8+ 52/117, 44.4%; CD20 + 27/117, 23.1%) [12]. Furthermore, another study has shown TILs were enriched in glioblastomas of the mesenchymal class [22]. Yang et al. have reported that an increased immune infiltrate of CD8+ cytotoxic T-cells predicts improved long-term survival in patients with glioblastoma [23]; however, this has not been replicated in many other studies. In our study, CD8 was found to be increased in methylated compared to unmethylated tumour cores; however, this study did not directly compare CD8 expression with patient survival.

A direct lineage of CD4+ T-cells and T-regulatory cells is CD3+ CD4+ T-cells. T-regulatory cells suppress the response of self-reactive T-cells and downregulate antitumour immunity. Tregs express the unique transcriptional repressor protein FOXP3 [24]. We observed a statistically significant difference in FOXP3 (*p* = 0.03) between MGMT methylated and unmethylated tumour core, although overall counts are low (MGMTuc = 7.02 [0.51] and MGMTmc = 7.94 [0.73]). In a recent study of 186 IDH-wild-type glioblastoma patients, consecutively treated with radiochemotherapy, the presence of FoxP3 + cells was associated with a better overall survival (*p* = 0.04; HR: 0.62 [95% CI: 0.4–0.98]) [25], although other studies have shown that FoxP3+ infiltrate is associated with tumour recurrence [26]. Additionally, in a study by Fecci et al., it has been shown that absolute counts of both CD4+ T-cells and CD4+ CD25+ FOXP3+ CD45RO + T-cells (i.e., Tregs) are reduced in patients with malignant glioma, yet there is an increased fraction of the CD4+ compartment [27]. In another cohort of 135 gliomas including 52 glioblastomas regardless of pathological type, the median survival was 43 months [(95% confidence interval [CI], 26.9—not available months) in patients whose tumours stained negatively for FoxP3. In contrast, the median survival duration was 19.2 months (95% CI, 13.8–34.0 months) in patients whose tumours contained FoxP3+ Tregs (*p* < 0.001). It must be noted that this finding did not account for the confounding influence of tumour grade on survival. In this cohort, patients with higher grade gliomas, who have shorter overall survival, were more likely to have greater Tregs infiltration than patients with lower-grade gliomas. However, when the group performed univariate Cox proportional hazards analysis within glioma pathological subtypes, the percentage of cells that stained positive for FoxP3 did not seem to correlate with survival duration [28]. The degree of variability in the available literature on the impact of T-regulatory cells on OS of glioma patients, alongside the increased FoxP3 levels in methylated compared to unmethylated tumour core, signifies the need for further investigation.

### 6.2. Immune Checkpoints

PD-L1 on glioblastoma cells interacts with PD-1 normally expressed on the surface of T-cells and this interaction may suppress T-cell activation. In our cohort, PD-L1 expression is statistically increased in MGMT methylated tumour cores compared with unmethylated tumour cores of GBMs (*p* = 0.025). CheckMate 143 also showed evidence of increased PD-L1 expression in a cohort 27/40 (68%) glioblastoma raising the possibility of PD-L1 inhibitor (nivolumab) use in clinical practice [10]. However, preliminary results, published in a World Federation of Neuro-Oncology Society (WFNOS) Meeting 2017 abstract, demonstrated a failure of PD-I inhibitor nivolumab to prolong overall survival of patients with recurrent glioblastoma, and this arm of the trial was prematurely closed [11]. One explanation for this lack of efficacy of nivolumab could be the lack of immune T-cells within GBM in line with observed comparatively low normalised mean count values of PD-1 (MGMTuc = 7.43 [0.44] and MGMTmc = 8.08 [0.73]) and in keeping with overall comparatively low T-cell associated proteins in our cohort, compared with PD-L1 (MGMTuc = 11.50 ([0.57] and MGMTmc = 10.62 [0.58]).

In addition, we observed higher overall mean normalised values of the checkpoint molecule B7-H3 (CD276) irrespective of methylation status; MGMT methylated (tumour core: 14.07 [1.02], MGMT methylated tumour margin: 12.70 [0.94]) compared to MGMT unmethylated GBM (tumour core: 12.35 [0.87], tumour margin: 12.52 [1.44]). We also noted a statistical increase of B7-H3 in MGMT methylated tumour core B7-H3 (CD276) against MGMT unmethylated tumour core (*p* = 0.023). A similar expression pattern has recently been reported by a study (*n* = 994) by Wang et al. who demonstrated B7-H3 upregulation in high-grade glioma and hence lower overall survival [29]. B7-H3 (CD276), which belongs to the B7 superfamily, has been shown to costimulate the proliferation of CD4+ and CD8+ T-cells, enhances the induction of cytotoxic T-cells, and stimulates interferon gamma production [30]. In comparison to PD-1 and CTLA-4 checkpoints, the mechanism of B7-H3 (CD276) in suppressing tumour development still remains unclear [31]. Considering the conclusions drawn by Wang et al. and the emerging evidence from this small-sample study, B7-H3 may be a potential therapeutic target that requires further investigation.

V-domain immunoglobulin suppressor of T-cell activation (VISTA), which is homologous to PD-L1, is a suppressor of T-cell activation, synergising with PD-1 [32, 33]. In addition, VISTA has also been shown to activate Tregs [34]. In our study, VISTA mean normalised counts were on the higher end of the normalised values' range in both MGMT methylated and unmethylated IDH-wild-type GBM; however, there was no significant difference between groups: MGMT methylated tumour core 11.94 [0.70], MGMT unmethylated tumour core 11.42 [0.77].

Although in our small study there was a statistically significant increase in CD4, CD14, CD68, CD8A, B7-H3, PDL-1, CD19, FOXP3, CD44, and STAT3 protein expression in methylated versus unmethylated GBM tumour core (see Table 2), we need to be cautions with these results due to the small sample size that requires further exploration and validation in larger cohorts using DSP.

## 7. Limitations

The small size of our cohort limits interpretation. In this study, the four main sources of variation in using DSP are nCounter quantification, ROI background, ROI size, and ROI cellularity [20]. Section-to-section proximity and alignment may lead to small fluctuations in raw, normalised, and relative nCounter data. In addition, section-to section variability may lead to decreased reproducibility of counts. Antibody cocktail lot variation may affect raw and positive protein tag normalization (ERCC normalization) nCounter counts. According to protocol, antibody cocktails are prepared fresh (i.e., weekly) which may lead to additional variation. In addition, pipetting variability and lot age can lead to differences in overall nCounter counts that can be corrected by housekeeping normalization.

Another disadvantage of DSP technology is that it only permits targeted discovery, and a selection of targets is required to be included in the cocktail of antibodies, rather than “pure discovery” [35]. Other than quantifying immune protein targets, this multiplex platform does not inform the mechanistic nature, properties, and function of the complex cellular and molecular pathways that take place in the ROI as these occur in space and time. NanoString GeoMx® Digital Spatial Profiling (DSP) technology produces high-dimensional multiplex data, which can complicate the normalization process and calls for caution and consistency when analysing the results, possibly in collaboration with computational scientists [20].

## 8. Conclusion

In our experience, careful consideration of the experimental design and normalization process, with simultaneous in-depth understanding of DSP normalization may optimise results. In this small study, there was a statistical increase in CD4, CD14, CD68, CD8A, B7-H3, PDL-1, CD19, FOXP3, CD44, and STAT3 protein expression in methylated versus unmethylated GBM tumour core; however, this requires larger cohort validation. Advanced multiplex immunooncological predictive biomarker analysis may underpin a future personalised medicine approach in the use of targeted immunotherapeutic agents in glioblastoma.

## Figures and Tables

**Table 1:** Mean (SD) log 2 housekeeping normalised values by location and MGMT status.

Immunooncology targets and controls	Tumour core		Tumour margin	
	Methylated	Unmethylated	Methylated	U nmethylated
	Mean (SD)	Mean (SD)	Mean (SD)	Mean (SD)
CD4	11.22 (0.65)	9.58 (1.01)	10.16 (0.84)	9.68 (0.99)
CD14	12.58 (1.61)	9.47 (1.46)	10.79 (1.07)	9.75 (1.31)
CD68	12.97 (1.00)	11.14 (0.80)	12.32 (1.14)	11.67 (1.29)
CD8A	8.30 (0.78)	6.90 (0.66)	8.06 (0.82)	7.70 (0.68)
B7-H3	14.07 (1.02)	12.35 (0.87)	12.70 (0.94)	12.52 (1.44)
PD-L1	11.50 (0.57)	10.62 (0.58)	11.47 (0.51)	11.65 (0.77)
CD19	7.77 (0.74)	6.66 (0.63)	7.47 (0.37)	7.67 (0.87)
FOXP3	7.94 (0.73)	7.02 (0.51)	7.75 (0.58)	7.88 (0.76)
CD44	15.58 (1.24)	13.30 (1.60)	13.51 (1.49)	14.80 (2.61)
STAT3 (phospho Y705)	9.41 (0.61)	8.64 (0.53)	8.52 (0.51)	9.06 (1.03)
CD45	9.19 (1.00)	7.93 (0.83)	8.25 (0.83)	8.83 (1.44)
Pan Cytokeratin	9.11 (0.67)	8.43 (0.31)	8.92 (0.30)	9.35 (0.82)
MS4A1.CD20.	7.26 (0.63)	6.47 (0.53)	7.15 (0.65)	7.50 (0.69)
CD45RO	8.29 (0.81)	7.48 (0.50)	7.88 (0.39)	8.28 (0.92)
S6	13.64 (0.30)	13.21 (0.39)	13.60 (0.32)	13.27 (0.46)
PD1	8.08 (0.73)	7.43 (0.44)	7.47 (0.72)	7.87 (0.79)
CD3	8.90 (0.61)	8.04 (1.02)	9.28 (0.70)	9.17 (1.00)
Beta-2 microglobulin	13.80 (1.12)	12.91 (0.53)	13.78 (0.69)	13.33 (1.02)
VISTA	11.94 (0.70)	11.42 (0.77)	11.80 (0.45)	12.13 (1.59)
Bcl2	10.22 (0.84)	9.70 (0.50)	9.93 (0.32)	10.51 (1.12)
GZMB	10.65 (0.47)	10.27 (0.60)	10.68 (0.68)	10.73 (0.46)
PTEN	11.29 (1.11)	10.56 (1.08)	11.13 (0.59)	11.99 (1.57)
Beta-catenin	16.19 (0.66)	16.61 (0.51)	16.53 (0.52)	17.65 (0.72)
CD56	18.40 (0.98)	17.73 (1.37)	19.26 (0.91)	19.15 (1.33)
Ki-67	11.72 (1.76)	10.81 (1.27)	10.83 (0.99)	9.96 (1.49)
STAT3	12.85 (1.44)	13.24 (0.87)	12.95 (0.83)	13.32 (0.89)
AKT	14.57 (0.68)	14.55 (0.59)	15.06 (0.56)	14.82 (1.00)
P-Akt	11.21 (0.61)	11.24 (1.46)	11.51 (0.95)	12.14 (1.16)

**Table 2:** *p* values for MGMT comparison in tumour core (column 2) and margin (column 3) and for comparing core against margin in methylated (column 4) and in unmethylated (column 5).

Immunooncology targets and controls	Tumour core	Tumour margin	Methylated	Unmethylated
	Methylated versus unmethylated	Methylated versus unmethylated	Core versus margin	Core versus margin
**CD4**	**0.005**	0.452	0.050	0.452
**CD14**	**0.006**	0.246	0.117	0.246
**CD68**	**0.007**	0.416	0.316	0.416
**CD8A**	**0.011**	0.383	0.617	0.383
**B7-H3**	**0.023**	0.793	0.087	0.793
**PD-L1**	**0.025**	0.639	0.875	0.639
**CD19**	**0.028**	0.614	0.406	0.614
**FOXP3**	**0.030**	0.722	0.577	0.722
**CD44**	**0.032**	0.451	0.068	0.451
**STAT3 (phospho Y705)**	**0.034**	0.396	^*∗*^0.048	0.396
CD45	*∗*0.041	0.358	0.125	0.358
Pan Cytokeratin	*∗*0.048	0.370	0.617	0.370
MS4A1/CD20	0.053	0.347	0.738	0.347
CD45RO	0.093	0.438	0.379	0.438
S6	0.100	0.218	0.928	0.218
PD1	0.121	0.390	0.205	0.390
CD3	0.144	0.830	0.259	0.830
Beta-2 microglobulin	0.147	0.423	0.981	0.423
VISTA	0.153	0.663	0.624	0.663
Bcl2	0.230	0.376	0.499	0.376
GZMB	0.274	0.317	0.155	0.317
PTEN	0.292	0.239	0.790	0.239
Beta-catenin	0.296	^*∗*^0.025	0.473	^*∗*^0.025
CD56	0.357	0.860	0.105	0.860
Ki-67	0.378	0.279	0.358	0.279
STAT3	0.634	0.619	0.841	0.619
AKT	0.946	0.644	0.207	0.644
P-Akt	0.978	0.353	0.505	0.353

^*∗*^Bold ^*∗*^Values statistically significant after Benjamini–Hochberg adjustment for False Discovery Rate at 0.1. ^*∗*^Values statistically significant if unadjusted but not statistically significant after Benjamini–Hochberg adjustment for False Discovery Rate at 0.1.

## Data Availability

Raw data obtained from specimen analysis are available from KMK upon request.
